# Expression of glycosaminoglycans in cirrhotic liver and hepatocellular carcinoma—a pilot study including etiology

**DOI:** 10.1007/s00216-022-04025-3

**Published:** 2022-03-28

**Authors:** Gábor Tóth, Domonkos Pál, Simon Sugár, Ilona Kovalszky, Katalin Dezső, Gitta Schlosser, László Drahos, Lilla Turiák

**Affiliations:** 1 MS Proteomics Research Group, Research Centre for Natural Sciences, Magyar tudósok körútja 2, H-1117 Budapest, Hungary; 2 Department of Inorganic and Analytical Chemistry, Budapest University of Technology and Economics, Szent Gellért tér 4, 1111 Budapest, Hungary; 3 Ph.D. School of Pharmaceutical Sciences, Semmelweis University, Üllői út 26, 1085 Budapest, Hungary; 4 1st Department of Pathology and Experimental Cancer Research, Semmelweis University, Üllői út 26, 1085 Budapest, Hungary; 5 MTA-ELTE Lendület Ion Mobility Mass Spectrometry Research Group, Eötvös Loránd University, Pázmány Péter sétány 1, 1117 Budapest, Hungary

**Keywords:** Glycosaminoglycan, Chondroitin sulfate, Heparan sulfate, Hepatocellular carcinoma, Liver cancer, Cirrhosis

## Abstract

Chronic liver diseases have both high incidence and mortality rates; therefore, a deeper understanding of the underlying molecular mechanisms is essential. We have determined the content and sulfation pattern of chondroitin sulfate (CS) and heparan sulfate (HS) in human hepatocellular carcinoma and cirrhotic liver tissues, considering the etiology of the diseases. A variety of pathological conditions such as alcoholic liver disease, hepatitis B and C virus infections, and primary sclerosing cholangitis were studied. Major differences were observed in the total abundance and sulfation pattern of CS and HS chains. For example, the *6-O-*sulfation of CS is fundamentally different regarding etiologies of cirrhosis, and a 2–threefold increase in HS *N-*sulfation/*O-*sulfation ratio was observed in hepatocellular carcinoma compared to cirrhotic tissues.

**Graphical abstract:**

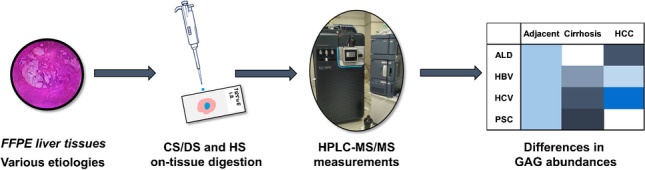

**Supplementary Information:**

The online version contains supplementary material available at 10.1007/s00216-022-04025-3.

## Introduction

Proteoglycans (PGs) are a group of biomolecules that contain at least one glycosaminoglycan (GAG) chain as a post-translational modification on a defined core protein. They can mainly be found in the extracellular matrix, on the cell surface, and in granules found in the cytoplasm, thus exerting essential signaling functions [1]. In healthy liver tissue, cell-surface heparan sulfate PGs are the primary type; however, chronic liver diseases can result in differential expression and structural alterations of other proteoglycan subclasses as well. Physicochemical properties and biological functions of PGs are strongly determined by the length and structure of GAG chains; therefore, it is worth investigating their organization between distinct biological conditions.

The two most prevalent classes of GAGs in tissues are chondroitin sulfate/dermatan sulfate (CS/DS) and heparan sulfate (HS). CS consists of alternating saccharide units of *N-*acetylgalactosamine (GalNAc) and glucuronic acid (GlcA), while HS is built up by *N-*acetylglucosamine (GlcNAc) and glucuronic/iduronic acid (IdoA). A very important step of GAG chain formation is sulfation, carried out by sulfotransferases [2]. The GalNAc residues in CS may be sulfated at the *4-OH* and/or *6-OH* positions, while GlcA may be sulfated at the *2-OH* position [3]. The variability of HS sulfation is a bit more complex. *N-*Acetylglucosamine *N-*deacetylase/*N-*sulfotransferase (NDST) enzymes act on given domains of GlcNAc residues to generate *N-*sulfation. Then, an epimerase acts on some GlcA residues, followed by possible *2-O-*sulfation of both IdoA and GlcA residues. Next, *6-O-*sulfotransferases exert their functions; then, finally, *3-O-*sulfation may also occur.

The GAG chains are responsible for cellular signaling and recognition, governed by the size and the sulfation pattern of the respective chains [4–6]. Alterations in the ratio of the differentially sulfated disaccharide building blocks may be descriptive of various diseases, e.g., sulfation pattern changes have been observed between healthy and cancerous tissues [7, 8].

HS chains isolated from hepatocellular carcinoma (HCC) are increased in size compared to those isolated from the non-cirrhotic peritumoral tissue [9]. Liver cirrhosis is accompanied by increased synthesis of connective tissue, together with the increase of CS GAGs. HCC also exhibits a selective increase in CS levels [8, 10]. This structural variability also appears on the level of proteoglycans influencing signal transduction. Decorin, a PG bearing either CS or DS chains, can serve as a tumor suppressor in liver cancer as it can affect multiple signaling pathways dysregulated in HCC [11]. Perlecan plays a central role in both physiologic and pathologic angiogenesis based on the high capacity of its HS chains to bind the two major proangiogenic factors, vascular endothelial growth factor and basic fibroblast growth factor [12]. Serglycin, the only intracellular proteoglycan, bears heparin and/or CS side chains that enable interaction with a variety of inflammatory mediators, such as proteases, cytokines, and growth factors [13, 14].

Most of the investigations nowadays targets fibrotic, cirrhotic, and hepatocellular carcinoma tissues without taking into consideration the etiology of the patients. However, etiology can be a crucial point in the molecular characteristics as proteoglycans can mediate the internalization of several viral species into the cells. Specifically, hepatitis C (HCV) and COVID-19 virus internalizations were shown to be influenced by cell-surface syndecans [15–17]. There are several examples of proteoglycans changing in liver diseases as well; however, those are seldom analyzed regarding their etiology. For example, syndecan-1 was shown to increase in cirrhosis compared to normal liver tissue, without correlation with etiology [18]. However, etiological correlations were observed in HCC tissues: compared to normal liver areas, there was a large increase of syndecan-1 levels in cirrhosis-associated HCC and a moderate increase in non-cirrhotic HCC. The same trend was observed for HCV-positive tissues as well [18].

Therefore, our primary aim was to carry out a pilot study to determine the impact of the etiology on the CS/DS and HS disaccharide composition in cirrhosis and HCC. This was performed through disaccharide analysis [3, 19, 20] using previously published HPLC–MS [21] methods enabling the analysis of disaccharides extracted from the surface of tissue sections. The resulting disaccharides have a characteristic sulfation pattern, which is descriptive of sulfation motifs of the original molecules.

## Materials and methods

### Selection of human samples

In this retrospective study, 11 explanted cirrhotic human livers without HCC were selected with the following etiologies: primary sclerosing cholangitis (PSC), hepatitis B virus (HBV), and hepatitis C virus (HCV). Five explanted cirrhotic human livers containing HCC with alcoholic liver disease (ALD), HBV, and HCV etiologies were selected and curated as one miscellaneous group of adjacent cirrhotic liver parenchyma. Finally, 14 hepatocellular carcinoma tissues with etiologies of ALD-associated cirrhosis (ALDC), HBV, and HCV were analyzed. All samples were carefully selected from the archives of the 1^st^ Department of Pathology and Experimental Cancer Research of Semmelweis University, Hungary. The properties of the full cohort are provided in Table S1.

### Preparation of FFPE tissue slices

Samples were fixed in 10% buffered formaldehyde and embedded into paraffin. Three-micrometer-thick sections were cut and stained with hematoxylin–eosin for diagnostic evaluation. Subsequently, 10-μm-thick non-stained paraffin-embedded sections were prepared from the paraffin-embedded blocks. Dewaxing before enzymatic digestion was performed by washing with xylene, ethanol–water mixtures, and 10 mM ammonium bicarbonate, respectively.

### On-tissue chondroitin sulfate digestion

Chondroitinase ABC digestion was performed based on a previously developed methodology [22]. Briefly, an aqueous digestion solution with the following composition was prepared: 20 mM Tris–HCl, 2.5 mM ammonium acetate, 10 mU/μL chondroitinase ABC (pH = 7.6). The used buffer ensures the selectivity of chondroitinase ABC towards CS/DS only. Data showing selectivity can be seen in Table S2. The enzyme solution was added in five cycles of 5-μL droplets onto the surface. The samples were incubated in a humidified box for 1 h at 37 °C in each cycle; then, an additional 43-h incubation was performed. The resulting disaccharides were extracted from the surface with 25 μL 0.3% ammonium hydroxide solution via 5 cycles of repeated pipetting. The samples were then dried down and stored at – 20 °C until further use. The structure and description of Δ^4,5^-unsaturated CS/DS disaccharides can be found in Table S3.

### On-tissue heparan sulfate digestion

Heparinase digestion was performed based on a previously developed methodology [22]. Briefly, an aqueous digestion solution with the following composition was prepared: 20 mM Tris–HCl, 2.5 mM Ca(OH)_2_, 10% glycerol, 0.5 mU/μL of heparin lyase I, 0.1 mU/μL of heparin lyase II, and 0.1 mU/μL of heparin lyase III. The enzyme solution was added in five cycles of 5-μL droplets onto the surface. The samples were incubated in a humidified box for 1 h at 37 °C in each cycle; then, an additional 43-h incubation was performed. The resulting disaccharides were extracted from the surface with 25 μL 0.3% ammonium hydroxide solution via 5 cycles of repeated pipetting. The samples were then dried down and stored at – 20 °C until further use. The structure and description of Δ^4,5^-unsaturated HS disaccharides can be found in Table S4.

### Sample cleanup with graphite solid-phase extraction (SPE)

A solid-phase extraction cleanup of the resulting CS and HS disaccharide mixtures was performed on Glygen graphite + C_18_ TopTips in a centrifuge pipet tip setup. The samples were applied in water, salts and contaminants were washed with water, then the disaccharides were eluted in 60:40 *v/v* H_2_O to acetonitrile (0.05% trifluoroacetic acid). The samples were then dried down and stored at − 20 °C until further use.

### Liquid chromatography-mass spectrometry

The HPLC–MS measurements were performed on a Waters Acquity I-class UPLC (Milford, MA) coupled to a Waters Select Series Cyclic Ion Mobility (Milford, MA) mass spectrometer. For the chromatographic separation of CS/DS and HS disaccharides, a self-packed GlycanPac AXH-1 capillary column (250 μm i.d.) was used with the ammonium formate salt gradient methods published before [23, 24]. In the low-flow ESI ion source, the capillary voltage was set to 1.9 kV, while the cone voltage was 20 eV and temperature was 120 °C. The HS disaccharides were measured in MS1 mode, the trap collision energy being 6 eV and the transfer being 3 eV. The CS/DS was measured in MS1 and MS/MS mode, where the monosulfated isomer pairs were fragmented with 32 eV in the transfer to determine stereochemistry.

### Data evaluation and interpretation

Peaks were integrated with the QuanLynx add-in of Waters MassLynx 4.2 software. Data visualization was done using R 4.0.5 in RStudio 1.4.1106 [25]; box plots were made using the ggplot package [26]. Effect sizes (based on Cohen’s *f*^2^) and subsequently sample sizes (for *α* = 0.05, and *1-β* = 0.9) were calculated using R.

### Chemicals and reagents

The Δ^4,5^-unsaturated chondroitin sulfate and heparan sulfate disaccharide standards and heparin lyase I-II-III enzymes were purchased from Iduron (Cheshire, UK). Crystalline ammonium formate, ammonium bicarbonate, chondroitinase ABC, and formic acid (FA) were purchased from Merck (Budapest, Hungary). LC–MS-grade solvents were purchased from VWR Hungary (Debrecen, Hungary). Glygen graphite + C_18_ TopTips were purchased from SunChrom GmbH (Friedrichsdorf Germany).

## Results

As chondroitin sulfate and dermatan sulfate are the main compounds of connective tissue, and chronic liver diseases are usually accompanied by connective tissue accumulation, it is expected that alterations may occur in both CS/DS quantity and structure. We have recently shown [24, 27] that compared to healthy liver, an increase in quantity and extent of sulfation is expected for both CS and HS GAGs. However, these findings were acquired regardless of the etiology which can have a great impact. Thus, we decided to take a closer look into these chronic liver diseases taking into account the etiology of the disease.

We compared the following tissues: cirrhotic liver tissues without HCC, cirrhosis-associated HCC, and adjacent cirrhotic liver parenchyma from HCC tissues. The etiologies for cirrhosis were the following: HBV and HCV infection, and primary sclerosing cholangitis. In the case of HCC tissues, the following etiologies were investigated: alcoholic liver disease (ALDC), HBV, and HCV infection. The etiologies of adjacent cirrhotic liver parenchyma were miscellaneous. First, the differences in total CS/DS and HS content of the tissues will be addressed; then, the alterations in the sulfation pattern of the respective GAGs will be discussed.

### Total CS/DS and HS content

**Fig. 1:**
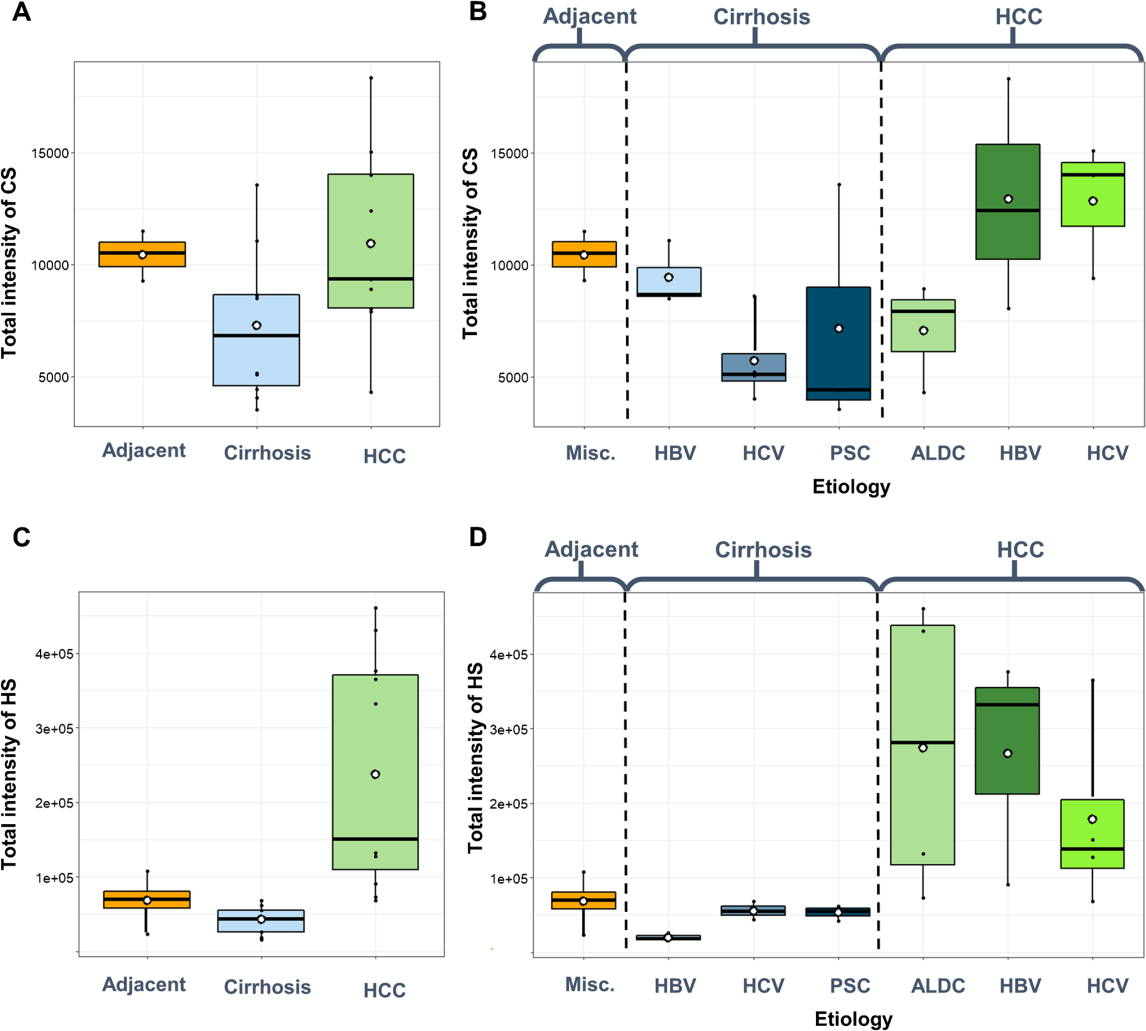
Total abundances of glycosaminoglycans in liver diseases. **A** Total CS/DS abundance without respect to the etiology. **B** Total CS/DS abundance with respect to the etiology. **C** Total HS abundance without respect to the etiology. **D** Total HS abundance with respect to etiology (HCC: hepatocellular carcinoma, ALDC: alcoholic liver disease–associated cirrhosis, HBV: hepatitis B virus, HCV: hepatitis C virus, PSC: primary sclerotizing cholangitis)

Without considering etiology, the total CS/DS content showed a small difference (Fig. 1A). The adjacent cirrhotic parenchyma and HCC samples had nearly the same CS/DS levels, while it was a bit lower in cirrhotic liver samples without HCC. However, these differences are relatively small, let alone capable of classification. Looking at the impact of the etiology of cirrhosis (Fig. 1B), we can see a relevant change between HBV- and HCV-associated cirrhotic samples, the latter exhibiting a CS/DS level 1.7 times less than in HBV-associated cirrhosis. However, this difference does not occur in HCC samples: the hepatitis viral origin resulted in CS/DS levels very similar to one another, but 1.8 times higher than ALDC etiology. Note, the total CS/DS levels of hepatitis virus–associated HCCs are higher, and that of ALDC-associated HCC is lower than the total CS/DS level of adjacent cirrhotic liver parenchyma tissues.

Compared to adjacent liver parenchyma, HS content was 1.5 times lower in cirrhotic liver samples without HCC, and it was on average 3.5 times larger in HCC. This resulted in a difference of 5.2 between cirrhosis without HCC and HCC (Fig. 1C). Note that the deviations in adjacent cirrhotic liver parenchyma and cirrhotic tissues are moderate, but the range of HS quantity in HCC is quite large (6.8 times difference between the smallest and largest values). This difference is, however, not due to the different etiologies, as a similar trend can be seen for HCC also in Fig. 1D. The large range of total HS data is in alignment with some recent research on syndecan-1 proteoglycan quantities as well [18]. Regarding total HS content in cirrhosis, the HBV-associated malfunction stands out from the others, having 2.6–2.8 times lower abundances (Fig. 1D).

The total CS/DS level is 1.7 times lower, while the total HS level is 2.8 times larger in HCV-associated cirrhosis than in HBV-associated cirrhosis. This opposite trend is not found in any other comparisons (Fig. 1B vs Fig. 1D).

To conclude, total CS/DS content is a bit larger in HCC than in cirrhosis (this aligns with our previous findings), and total HS content is more than 6 times larger on average in HCC. HBV and HCV infection-associated cirrhosis show a substantial difference; CS/DS levels are higher and HS levels are lower in HBV-associated cirrhosis.

### Structural variability of glycosaminoglycans—differences in sulfation pattern

For deciphering structural differences, we analyzed the sulfation pattern as described by the relative abundance of the respective.

#### Chondroitin sulfate/dermatan sulfate sulfation

**Fig. 2:**
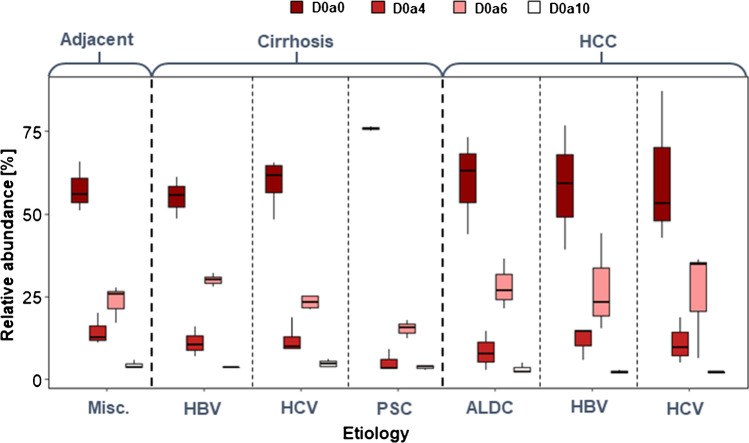
Sulfation pattern of CS/DS in liver diseases concerning etiology (HCC hepatocellular carcinoma, ALDC: alcoholic liver disease–associated cirrhosis, HBV: hepatitis B virus, HCV: hepatitis C virus, PSC: primary sclerotizing cholangitis)

Comparing HCC and the adjacent cirrhotic liver parenchyma, we can conclude that there is no detectable difference in terms of sulfation pattern, but the range of the disaccharide ratios increased by a factor of 2 in HCC, approximately (Fig. 2). This indicates that the increasing interpatient variability mentioned in “Total CS/DS and HS content” is also present in the structure of the expressed GAGs, not only in their total amount (Figs. 1 and  2).

The CS/DS sulfation pattern showed considerable differences in any pairwise comparisons of cirrhotic groups (Fig. 2). HBV- and HCV-associated cirrhosis resulted in a similar sulfation pattern to adjacent cirrhotic liver parenchyma. However, there is a remarkable difference between these two in the ratio of the D0a6 disaccharide. Besides, the levels of D0a4 disaccharides are higher than in the other two types of cirrhosis. The sulfation of CS/DS chains in PSC-associated cirrhosis is completely different from the other groups. The ratio of D0a0 disaccharide is 1.3–1.5 times larger, and the ratios of the monosulfated (D0a4 and D0a6) disaccharides are lower than those in any other cirrhotic samples (Fig. 2).

The *6-O/4-O*-sulfation ratio also shows some interesting results (Table 1). All the samples showed a higher *6-O* ratio than the adjacent cirrhotic liver parenchyma, ALDC-associated HCC, and PSC-associated cirrhosis having the largest.

**Table 1:** Sulfation characteristics of CS chains. The 6S/4S ratio is the quotient of the intensities of D0a6 and D0a4 disaccharides. The change in overall CS sulfation shows the change in the average number of sulfate groups per disaccharide building block compared to that in the tumor-adjacent cirrhotic liver parenchyma

Chronic liver disease	Etiology	6S/4S ratio	Change in overall CS sulfation
Tumor-adjacent cirrhotic liver parenchyma	Miscellaneous	1.61	-
Cirrhosis without HCC	HBV	2.69	1.03
Cirrhosis without HCC	HCV	1.86	0.97
Cirrhosis without HCC	PSC	2.94	0.59
Hepatocellular carcinoma	ALDC	3.37	0.92
Hepatocellular carcinoma	HBV	2.35	0.93
Hepatocellular carcinoma	HCV	2.31	0.87

The ratio of the doubly sulfated disaccharide (D0a10) is always the smallest, except in PSC-associated cirrhosis, and its mean value is not affected by the etiology. However, a large difference was observed in the level of D0a10 between HCV-associated cirrhosis and the two virus-associated HCC groups.

Looking at the absolute intensities of CS/DS disaccharides, the same trends were observed as discussed above for relative abundance (Fig. S1). However, the standard deviations of the measured values for PSC-associated cirrhosis and all the HCC groups are larger due to the deviations in total CS/DS quantities.

The overall sulfation of CS/DS chains in HCC shows an 8–13% decrease compared to the respective tumor-adjacent cirrhotic parenchyma, and no effect of etiology could be observed. This was not the case for the cirrhotic samples: in HBV- and HCV-associated cirrhosis practically, the same rate of sulfation was observed as in the tumor-adjacent cirrhotic tissues, but a 41% undersulfation in PSC-associated cirrhosis was detected (Table 1).

To conclude, PSC-associated cirrhosis results in a completely different sulfation pattern from any other liver malfunctions. *6-O*-sulfation is predominant in all types of cirrhosis and shows considerable differences between each pairwise comparison of cirrhotic groups, resulting in an interesting interchange between the D0a0 and D0a6 components.

#### Heparan sulfate sulfation

**Fig. 3:**
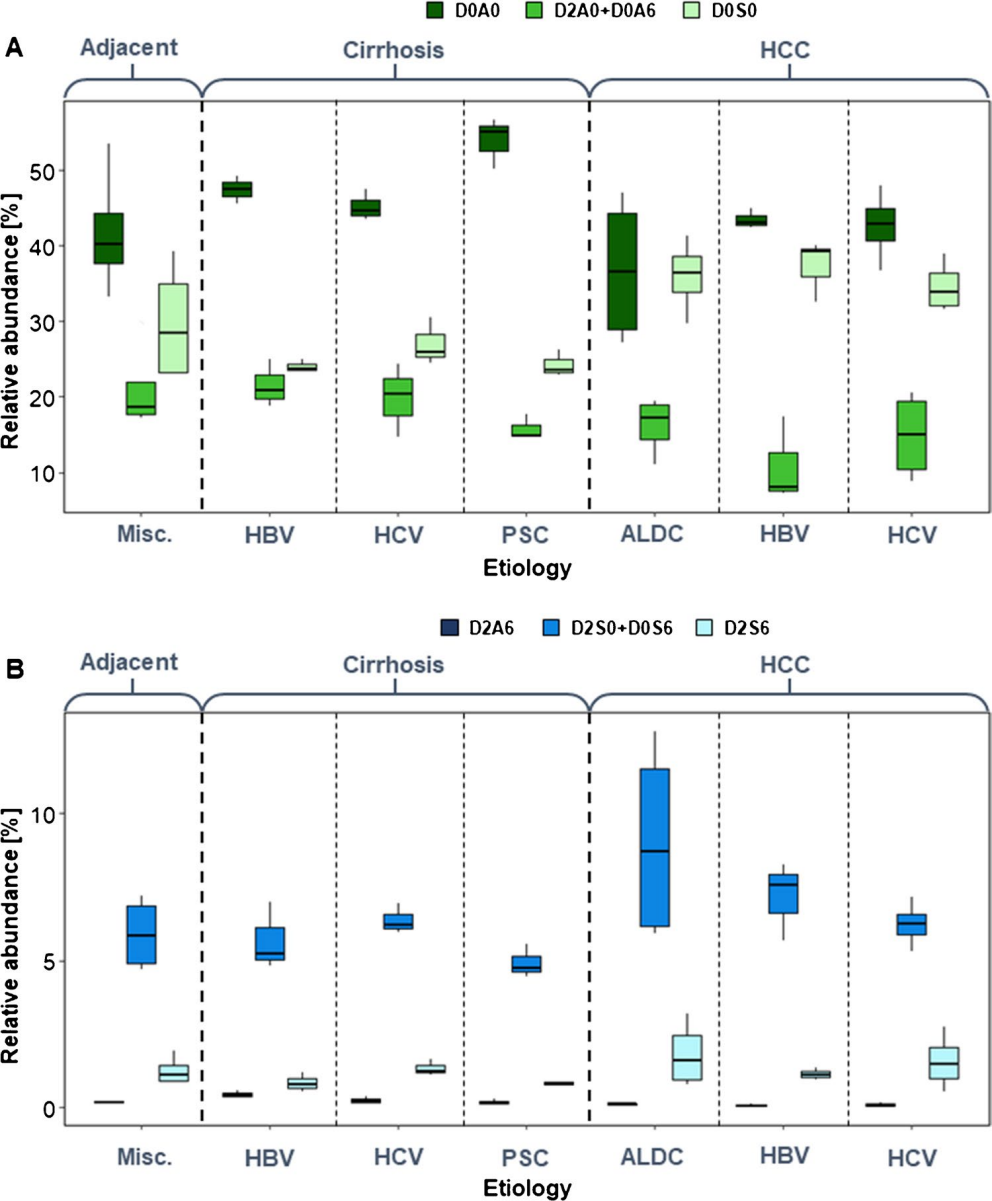
Sulfation pattern of heparan sulfate in liver diseases concerning etiology. **A** Non-sulfated and monosulfated HS disaccharides. **B** Doubly and triply sulfated HS disaccharides (HCC hepatocellular carcinoma, ALDC: alcoholic liver disease–associated cirrhosis, HBV: hepatitis B virus, HCV: hepatitis C virus, PSC: primary sclerotizing cholangitis)

Regarding sulfation motifs of HS, non-sulfated and monosulfated disaccharide building blocks dominate in most cases (Fig. 3). The doubly and triply sulfated disaccharides show lower abundance and smaller changes as well (Fig. 3B).

In general, cirrhotic tissues contain more non-sulfated (D0A0) and less monosulfated (D0S0 and D2S0/D0S6) disaccharides making the extent of sulfation lower than in HCC. The doubly and triply sulfated disaccharides do not show exact trends across cirrhosis and cancer (Fig. 3B). In most tissue samples, the quantity of the doubly *O*-sulfated disaccharide building block (D2A6) is very low (Fig. 3B). However, its ratio in HBV-associated cirrhosis is 1.9–6.0 times larger than that in any other investigated condition.

Compared to adjacent cirrhotic liver parenchyma, the extent of sulfation of the cirrhotic tissue is smaller. It differs most for PSC-associated cirrhosis, and the sulfation pattern was observed from the other etiology groups (see similar results for CS/DS chains in “Chondroitin sulfate/dermatan sulfate sulfation”). Besides, the ratio of the mono-*O*-sulfated components (D2A0/D0A6) shows the same trend as the D0a4 CS disaccharide. This similar trend might be subject to further investigation since there is no direct structural correlation between the given disaccharides.

The pairwise comparisons of HBV- and HCV-associated cirrhosis and HCC, respectively, show that the expression of D0S0 disaccharide is 1.3–1.6 times higher in HCC. This implicates that in HCC, *N*-sulfation is more dominant: the *N*-sulfated/*O*-sulfated ratio is 1.9 times higher for the monosulfated components (D0S0 vs D2A0/D0A6) and 3.4 times higher for the doubly sulfated components (D2S0/D0S6 vs D2A6). *N/O* ratios are listed in Table 2.

**Table 2:** Sulfation characteristics of HS chains. The monosulfated *N/O* ratio is the quotient of the intensities of D0S0 and D2A0 + D0A6 disaccharide intensities, while the disulfated *N/O* ratio is the quotient of the D2S0 + D0S6 and D2A6 disaccharide intensities. The change in overall HS sulfation compared to tumor-adjacent cirrhotic liver parenchyma shows the change in the average number of sulfate groups per disaccharide building block

Chronic liver disease	Etiology	Monosulfated *N/O* ratio	Disulfated *N/O* ratio	Change in overall HS sulfation
Tumor-adjacent cirrhotic liver parenchyma	Miscellaneous	1.42	32.01	-
Cirrhosis without HCC	HBV	1.11	12.38	0.91
Cirrhosis without HCC	HCV	1.37	26.23	0.96
Cirrhosis without HCC	PSC	1.54	25.87	0.79
Hepatocellular carcinoma	ALDC	2.22	70.03	1.14
Hepatocellular carcinoma	HBV	3.43	93.05	0.99
Hepatocellular carcinoma	HCV	2.33	58.92	1.00

Looking at the absolute intensities of HS disaccharides, the same trends were observed as discussed above for relative abundance (Fig. S2). However, smaller standard deviations were observed in adjacent cirrhotic liver parenchyma and all the cirrhosis groups, while those for HCC groups were considerably larger in most cases.

The sulfation rate of HS is lower in cirrhosis than in HCC and HCC-adjacent cirrhotic parenchyma. The etiology has a noteworthy effect on cirrhosis: HS chains in tissues with HCV etiology show 5.5% higher, while those in tissues with PSC etiology showed a 13.2% lower overall sulfation compared to the HBV-associated cirrhosis (Table 2). In HCCs with virus-associated etiology and the respective tumor-adjacent cirrhotic tissues, no difference was observed in the overall rate of sulfation, and increased sulfation of 14% in ALD-cirrhosis-associated HCC was detected (Table 2).

### Proteoglycan levels in cirrhosis

We have further analyzed the cirrhotic samples using shotgun proteomics to determine if the changes causing differences in total GAG levels can be attributed to changes in proteoglycan core protein expression levels. We found that none of the identified proteoglycans showed statistically significant changes in any pairwise group comparisons. For details in proteomics methodology and results, please see Table S5.

## Discussion

Disregarding etiology, the total CS/DS contents of cirrhotic and HCC samples are in line with our previous results [10, 24]. The sulfation of CS/DS chains in HCC was similar to that in the tumor-adjacent cirrhotic liver parenchyma, and no impact of etiology was observed (Fig. 2).

In contrast, striking differences related to etiology were observed in cirrhotic liver samples. The ratio of *6-O*-sulfated disaccharide (D0a6) varied up to twofold depending on the etiology of liver disease and decreased D0a6 was associated with increased D0a0 levels. HBV- and HCV-associated cirrhosis showed similar D0a6 levels to adjacent cirrhotic liver parenchyma (but a large difference was observed between these two), and PSC resulted in the lowest D0a6 ratio.

The *4-O*-sulfation showed much smaller differences, mainly PSC-associated cirrhosis had lower levels of D0a4. The etiology-related HCC data are in agreement with literature findings [28, 29]; however, to the authors’ knowledge, there has not been any study indicating etiology-related differences in GAG composition in the case of cirrhosis. The changes in D0a6 content may be attributed to the different levels of inflammation present in the livers, *6-O*-sulfation on CS/DS has been shown to have anti-inflammatory effects [28], and it is commonly accepted that the development of tumors is tightly linked to chronic low-grade inflammation [30]. Another mechanism that is involved in tumor progression is an increase in the growth factor, cytokine, and chemokine levels synthesized by tumor-associated macrophages. Extracellular chondroitin-6-sulfate is in interaction with CD44 proteoglycan and TLR 2, 4, and 9, and HARE receptors, thus reducing the expression of pro-inflammatory cytokines (e.g., interleukins 6 and 12) and increasing the synthesis of anti-inflammatory markers (e.g., interleukin-10 and TGFß) in the cytoplasm [28]. Another relevant fact is that an increased amount of *6-O*-sulfation has been reported to characterize the tissue remodeling connected to fibrosis [31]. This is contradictory to our findings since we observed decreased sulfation on fibrosis-related samples (Fig. 2). Therefore, a more detailed investigation is advised.

In the literature, there is controversy regarding how the structure of heparan sulfate changes during malignant transformation. For example, Nakamura reported that HS obtained from primary hepatocellular carcinoma was undersulfated compared to normal liver tissue [32]; however, Kovalszky et al. have observed a modest but significant decrease in *6-O*-sulfation and an increase in *3-O*-sulfation, while total HS sulfation levels did not differ between normal human liver and HCC [33].

In HS chains, we observed a shift between *N*-sulfated and *O*-sulfated regions between cirrhotic and HCC tissues. Ignoring the etiology, the ratio of the *N*-sulfated/*O*-sulfated domains increased on average 1.9-fold in the case of monosulfated disaccharides and 3.4-fold in the case of the doubly sulfated domains during malignant transformation. Etiology also had an impact on sulfation positions. HBV-associated cirrhosis resulted in the lowest *N/O* ratio both for monosulfated and disulfated disaccharides. HVC- and PSC-associated cirrhosis resulted in similar differences between *N*- and *O*-sulfation, PSC being larger. In the case of HCC, however, HBV etiology resulted in much higher *N-*sulfation than HCV- and ALDC-associated HCC (Fig. 3). Unfortunately, the extent and mechanism of *N*-sulfation pattern regulation are yet unknown. However, the lengths of the *N*-sulfated domains correlate with the concentration of the sulfate donor to NDST enzymes (3′-phosphoadenosine-5′-phosphosulfate, PAPS) [34]. The action of the NDST enzyme is described to be opposite to that of the EXT1/EXT2 polymerase complex [35]. NDST1 can bind to EXT2, and the *N*-sulfation degree is affected by the level of EXT1 and EXT2 expression [36].

Etiology has a moderate effect on the overall rate of sulfation of both CS/DS and HS chains. A direct correlation could only be observed in PSC-associated cirrhosis, as both CS/DS and HS suffered substantial undersulfation. HBV etiology caused a 5–6% higher rate of CS/DS sulfation than HCV etiology, and the opposite difference could be observed in the rate of HS sulfation in cirrhosis. However, between HBV- and HCV-associated HCC, no difference in the overall rate of HS sulfation was observed. Alcoholic liver disease resulted in the oversulfation of HS in HCC.

PSC etiology caused a completely different sulfation pattern of both CS/DS and HS chains in comparison with other cirrhotic conditions. One of the possible reasons is that one of the investigated samples was fibrotic tissue and another one was fibrosis-cirrhosis transformation. Therefore, it can dominate the GAG composition and cause a large standard deviation of the data. However, we observed practically the smallest relative standard deviations in the sulfation patterns and the total HS content of the PSC samples compared to others; only the total CS/DS quantity showed large deviations. It suggests that the total amount of CS/DS chains changes during fibrosis-cirrhosis transformation but other GAG characteristics remain constant. Besides, one of the HCC samples was derived from HBV-associated fibrosis and had a lower rate of sulfation on CS/DS and HS chains. Therefore, the fibrotic nature has an enormous impact on the GAG composition and should be taken into consideration when collecting samples.

Most of the HCC samples belonged to pathological grade 2, but one grade 1 and three grade 3 samples were also included in the cohort. Thus, we addressed if HCC progression had a direct effect on GAG composition. No obvious differences were observed, but in the future, it would be worth investigating a larger, grade-based cohort, as it has already been shown for other types of cancer that radical changes can occur in sulfation with cancer progression [7].

Proteoglycan core protein quantification with shotgun proteomics showed that the proteoglycan levels are not statistically different between the cirrhotic samples with distinct etiology. This finding is in alignment with a recent publication, where syndecan-1 levels were shown to be the same in cirrhotic tissues with different etiology [18]. However, it is interesting to see that no proteoglycans were expressed differentially, since it is well-known that PG expression can show large changes in cancer, including that of the liver [37, 38]. This leads to potential targeted proteomics and molecular biology-validated investigations to thoroughly analyze the effects of etiology on PG expression in both cirrhosis and HCC.

Finally, a desired sample size estimation was carried out to give the basis for a future large-scale study based on the variance in the data. The desired minimum sample size was calculated for each disaccharide in each sample group to discriminate the given group from the others. The conclusion it holds is that the ideal minimum sample size for each group would be ca. 35, although investigating a minimum of 20 samples/group could already result in substantial differentiation. The results are shown in detail in Table S6.

## Conclusions

Large variability in glycosaminoglycan content and sulfation structure was observed among cirrhotic livers without HCC, HCC, and tumor-adjacent cirrhotic liver parenchyma tissues. The total quantity of CS/DS is slightly higher in HCC and adjacent tissue than in cirrhosis, and the etiology strongly determines the extent of *6-O*-sulfation. The total quantity of HS was ca. 6 times higher in HCC and ca. 1.5 times higher in adjacent tissue than in cirrhosis. *N*-sulfation was preferred in HCC. PSC resulted in highly decreased sulfation of both CS/DS and HS chains. Although there are several considerable differences in disaccharide building block levels, the large interpersonal variability of GAG levels makes the interpretation of the HCC data difficult. Further large-scale prospective studies are necessary to validate these findings. Therefore, a study with at least 35 samples/group is to be performed in the future to obtain satisfactory statistical power to determine the significant changes. Samples with fibrosis and HCC having distinct pathological stages and grades, respectively, should also be taken into the aims of future investigations.

## Supplementary Information

Below is the link to the electronic supplementary material.

Supplementary file1 (PDF 511 KB)

Supplementary file2 (XLSX 14 KB)

## Data Availability

Not applicable.
