# Supplementary material for: Expression of glycosaminoglycans in cirrhotic liver and hepatocellular carcinoma—a pilot study including etiology
Source: Anal Bioanal Chem. 2022 Mar 28;414(13):3837–46. doi: 10.1007/s00216-022-04025-3 (PMC8958808; doi:10.1007/s00216-022-04025-3)
Supplement: Supplementary file 1 — Supplementary file1 (PDF 511 KB) [file 216_2022_4025_MOESM1_ESM.pdf]

*Supplementary material to the article:*

# Expression of glycosaminoglycans in cirrhotic liver and hepatocellular carcinoma – A pilot study including etiology

Gábor Tóth[a,b], Domonkos Pál[a], Simon Sugár[a,c], Ilona Kovalszky[d], Katalin Dezső[d], Gitta Schlosser[e], László Drahos[a], Lilla Turiák[a,*]

[a]MS Proteomics Research Group, Research Centre for Natural Sciences, Magyar tudósok körútja 2., H-1117 Budapest, Hungary

[b]Department of Inorganic and Analytical Chemistry, Budapest University of Technology and Economics, Szent Gellért tér 4., H-1111 Budapest, Hungary

[c]Ph.D. School of Pharmaceutical Sciences, Semmelweis University, Üllői út 26, H-1085, Budapest, Hungary

[d]1st Department of Pathology and Experimental Cancer Research, Semmelweis University, Üllői út 26, H-1085, Budapest, Hungary

[e]MTA-ELTE Lendület Ion Mobility Mass Spectrometry Research Group, Eötvös Loránd University, Pázmány Péter sétány 1, H-1117, Budapest, Hungary

***Corresponding author**

Lilla Turiák
Research Centre for Natural Sciences
Magyar tudósok körútja 2., H-1117, Budapest, Hungary
Email: turiak.lilla@ttk.hu
Tel: +36 1 382 6548

# Table of contents

**Table S-1.** Details of the samples selected for the analysis – uploaded separately as an .xlsx file

**Table S-2.** Intensities of the D0a0 disaccharide with and without hyaluronan (HA) digestion preceeding the CS/DS digestion.

**Table S-3.** Structure, nomenclature, and m/z values of the CS/DS disaccharides investigated. D0a4 and D0a6 are positional isomers and are distinguished via MS/MS measurements in the present study.

**Table S-4.** Structure, nomenclature, and *m/z* values of the HS disaccharides investigated.

**Figure S-1.** Sulfation pattern of CS/DS in liver diseases concerning etiology shown by absolut intensities of disaccharides. (Cirr: cirrhosis, HCC: hepatocellular carcinoma, ALDC: alcoholic liver disease associated cirrhosis, HBV: hepatitis-B virus, HCV: hepatitis-C virus, PSC: primary sclerotizing cholangitis)

**Figure S-2.** Sulfation pattern of heparan sulfate in liver diseases concerning etiology shown by absolut intensities of disaccharides. **A:** non-sulfated and monosulfated HS disaccharides, **B:** doubly and triply sulfated HS disaccharides. (Cirr: cirrhosis, HCC: hepatocellular carcinoma, ALDC: alcoholic liver disease associated cirrhosis, HBV: hepatitis-B virus, HCV: hepatitis-C virus, PSC: primary sclerotizing cholangitis)

**Table S-5.** Kruskal-Wallis *p*-values of proteoglycans among the cirrhotic groups according to shotgun proteomics experiments – a detailed description of the proteomics methods is also provided.

**Table S-6.** Estimation of minimum sample size for a future large-scale study based on HS and CS/DS sulfation pattern.

# Checking the CS/DS digestion specificity of Chondroitinase ABC

We performed analysis on 4-4 serial sections of liver tissues with or without preceeding HA digestion. We found that no bias is caused by not performing the hyaluronan (HA) digestion. Moreover, the variance of D0a0 intensities proved to be larger when HA digestion was performed before CS digestion (Table S-2).

*The method for HA digestion ws the following:*

Five cycles 5 µL droplets of testicular hyaluronidase (Merck Sigma, Hungary) enzyme solution (0.16 TRU/µL hyaluronidase in the presence of 2 M ammonium acetate and 10% glycerol) was applied on the chosen spots. The resulting HA disaccharides were extracted four times by 0.3% ammonium hydroxide solution and the slides were dried. After this, CS/DS digestion was performed on the same spot as described in the Materials and Methods of the main body.

**Table S-2.** Intensities of the D0a0 disaccharide with and without hyaluronan (HA) digestion preceeding the CS/DS digestion.

| | **HA digested sample** | **non-HA digested sample** |
|---|---|---|
| **1** | 13915 | 10239 |
| **2** | 8969 | 11169 |
| **3** | 12379 | 12313 |
| **4** | 15497 | 10375 |
| **Average** | 12690 | 11024 |
| **relative standard deviation** | 19.03% | 7.48% |
| **t-test *p*-value** | 0.3262 | |

# Investigated disaccharides

**Table S-3.** Structure, nomenclature, and m/z values of the CS disaccharides investigated. D0a4 and D0a6 are positional isomers and are distinguished via MS/MS measurements in the present study.

| Chemical structure | Traditional name | Lawrence code | m/z (-) mode | MS/MS transition |
|---|---|---|---|---|
| 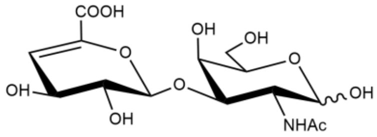 | ΔHexA-GalNAc | **D0a0** | 378.1 | - |
| 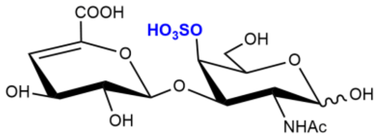 | ΔHexA-GalNAc4S | **D0a4** | 458.1 | 300.1 |
| 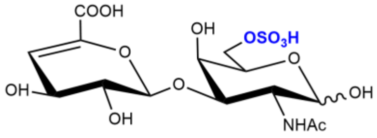 | ΔHexA-GalNAc6S | **D0a6** | 458.1 | 282.1 |
| 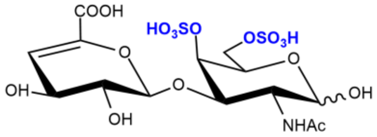 | ΔHexA-GalNAc4S6S | **D0a10** | 538.1 | - |

**Table S-4.** Structure, nomenclature, and *m/z* values of the HS disaccharides investigated.

| Chemical structure | Traditional name | Lawrence code | m/z (-) mode |
|---|---|---|---|
| 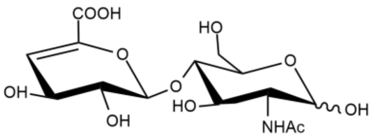 | ΔHexA-GlcNAc | **D0A0** | 378.1 |
| 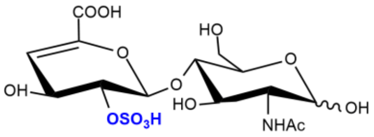 | ΔHexA2S-GlcNAc | **D2A0** | 458.1 |
| 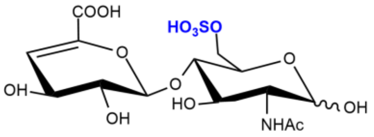 | ΔHexA-GlcNAc6S | **D0A6** | 458.1 |
| 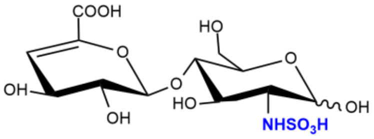 | ΔHexA-GlcNS | **D0S0** | 416.1 |
| 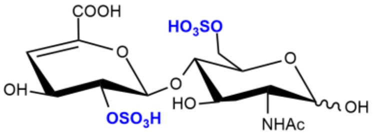 | ΔHexA2S-GlcNAc6S | **D2A6** | 538.1 |
| 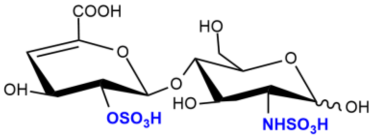 | ΔHexA2S-GlcNS | **D2S0** | 496.1 |
| 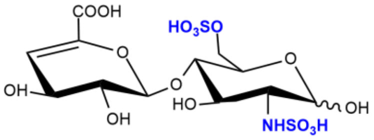 | ΔHexA-GlcNS6S | **D0S6** | 496.1 |
| 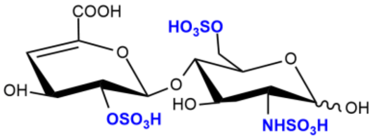 | ΔHexA2S-GlcNS6S | **D2S6** | 576.1 |

# Sulfation pattern of CS and HS disaccharides as shown by absolute intensities

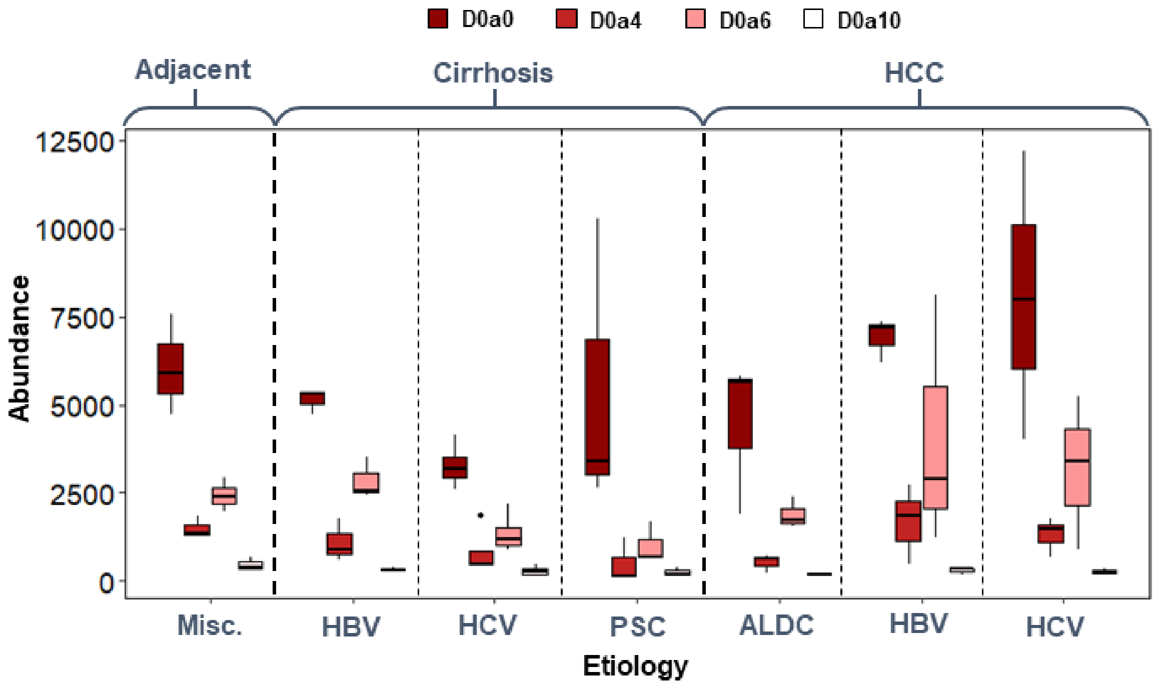


**Figure S-1.** Sulfation pattern of CS/DS in liver diseases concerning etiology shown by absolut intensities of disaccharides. (Cirr: cirrhosis, HCC: hepatocellular carcinoma, ALDC: alcoholic liver disease associated cirrhosis, HBV: hepatitis-B virus, HCV: hepatitis-C virus, PSC: primary sclerotizing cholangitis)

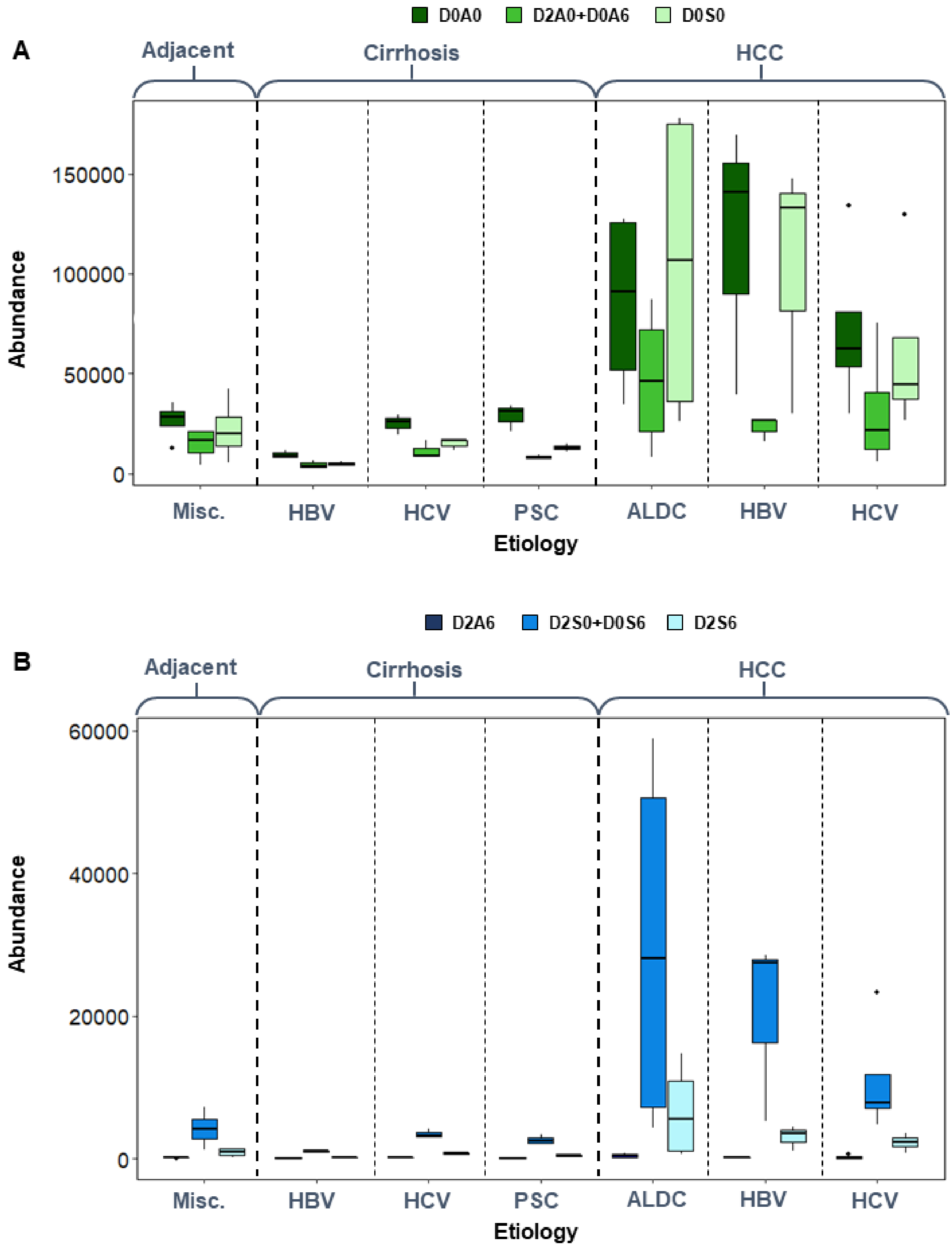


**Figure S-2.** Sulfation pattern of heparan sulfate in liver diseases concerning etiology shown by absolut intensities of disaccharides. **A:** non-sulfated and monosulfated HS disaccharides, **B:** doubly and triply sulfated HS disaccharides. (Cirr: cirrhosis, HCC: hepatocellular carcinoma, ALDC: alcoholic liver disease associated cirrhosis, HBV: hepatitis-B virus, HCV: hepatitis-C virus, PSC: primary sclerotizing cholangitis)

# Shotgun proteomics experiments

## 1. On-surface digestion

After CS and HS surface digestion, the same tissue spots were used for proteomic digestion. First, the proteins were reduced using RapiGest and DTT in 5 µL of 20% glycerol for 20 minutes at 55 °C, then alkylated using IAA in 5 µL of 25 mM ammonium bicarbonate (ABC) puffer and 20% glycerol for 20 minutes at room temperature in the dark. The digestion was done cyclically, each one lasting for 40 minutes at 37 °C in a humidified box, 5 cycles in total. In the first two cycles, LysC-Trypsin mixture was added in a 1:25 ratio, in 5 µL 50 mM ABC, and 20% glycerol. Subsequently, in the last three cycles, Trypsin was added in a 1:10 ratio, in 5 µL 50 mM ABC, and 20% glycerol. After digestion, proteins were extracted from the surface with 5x5 µL of 10% acetic acid. Peptide extracts were then dried down, and clean-up was performed using C18 spin columns (Thermo Scientific) using the manufacturer's protocol. The cleaned peptide extracts were dried down and stored at -20°C for further usage.

## 2. Mass spectrometry and chromatography analysis

Samples were dissolved in 8 µL solvent (98% water, 2% acetonitrile, and 0.1% formic acid) out of which 6 µL was subjected to nanoLC-MS/MS analysis using a Dionex Ultimate 3000 RSLC nanoLC (Dionex, Sunnyvale, CA, USA) coupled to a Bruker Maxis II Q-TOF (Bruker Daltonik GmbH, Bremen, Germany) via CaptiveSpray nanoBooster ionization source. Peptides were separated on an Acquity M-Class BEH130 $C_{18}$ analytical column (1.7µm, 75µm x 250mm Waters, Milford, MA) using gradient elution (isocratic hold at 4% for 11 min, then elevating B solvent content to 25% in 75 min, and 40% in 15 min) following trapping on an Acclaim PepMap100 C18 (5µm, 100µm x 20mm, Thermo Fisher Scientific, Waltham, MA) trap column. Solvent A consisted of water + 0.1% formic acid, while Solvent B was acetonitrile + 0.1% formic acid. Spectra were collected using a fixed cycle time of 2.5 sec and the following scan speeds: MS spectra at 3 Hz, while CID was performed on multiply charged precursors at 16 Hz for abundant ones and at 4 Hz for low abundance ones. Internal calibration was performed by infusing sodium formate and data were automatically recalibrated using the Compass Data Analysis software 4.3 (Bruker Daltonik GmbH, Bremen, Germany).

## 3. Data analysis

Database search was performed with Byonic 3.8 software. 10ppm internal and 20 ppm external mass accuracy, 2 missed cleavages, carbamidomethylation of cysteines as fixed modification, deamidation (NQ) and oxidation (M) as variable modifications) and proteins were identified using 1% FDR limit. A focused FASTA file was created from the proteins identified in all the samples and that was used as the database for label-free quantitation using MaxQuant 2.0.3.0 using default setting for Bruker QTOF [1]. The MaxQuant output was then loaded into Perseus 1.6.5.0, missing values were imputed from a normal distribution with the default settings for the whole matrix (down shift of 1.8 and width of 0.3). Statistical analysis was then performed, Kruskal-Wallis test.

## 4. Results

The proteoglycans identified in the samples were specifically searched for if they showed significant quantity differences among the samples. The shotgun proteomics analysis determined, that no proteoglycans exerted significant changes, thus the difference in GAG composition is due to the alteration in the polysaccharide synthesis. The Kruskal-Wallis p-values of the quantified proteoglycans are shown in Table S-4.

**Table S-5.** Kruskal-Wallis *p*-values of proteoglycans among the cirrhotic groups according to shotgun proteomics experiments.

| Uniprot ID | Proteoglycan | KW *p*-values |
|---|---|---|
| P20774 | Mimecan | 0.3011 |
| P07585 | Decorin | 0.3454 |
| P21810 | Biglycan | 0.5210 |
| P51888 | Prolargin | 0.5407 |
| Q99715 | Collagen alpha-1(XII) chain | 0.7365 |
| P16070 | CD44 | 0.7423 |
| P98160 | Perlecan | 0.7466 |
| P13611 | Versican | 0.7845 |
| O00468 | Agrin | 0.7926 |
| P51884 | Lumican | 0.8214 |

1. J. Cox, M. Mann, MaxQuant enables high peptide identification rates, individualized p.p.b.-range mass accuracies and proteome-wide protein quantification, Nature Biotechnology. 26 (2008). 1367-1372. DOI: 10.1038/nbt.1511.

**Table S-6.** Estimation of minimum sample size for a future large-scale study based on HS and CS sulfation pattern.

| HS sulfation pattern | | | | | | |
|---|---|---|---|---|---|---|
| | **D0A0** | **D2A0/D0A6** | **D0S0** | **D2A6** | **D2S0/D0S6** | **D2S6** |
| **Adjacent** | 12 | 14 | 11 | 353 | 42 | 33 |
| **Cirr_HBV** | 417 | 75 | 1341 | 41 | 75 | 119 |
| **Cirr_HCV** | 308 | 32 | 109 | 32 | 393 | 179 |
| **Cirr_PSC** | 114 | 276 | 378 | 52 | 306 | 2957 |
| **HCC_ALDC** | 8 | 34 | 30 | 278 | 5 | 5 |
| **HCC_HBV** | 728 | 23 | 65 | 203 | 54 | 287 |
| **HCC_HCV** | 41 | 14 | 63 | 106 | 114 | 8 |

| | | |
|---|---|---|
| Min 20 samples discriminate between | **8** | comparisons |
| Min 25 samples discriminate between | **9** | comparisons |
| Min 30 samples discriminate between | **10** | comparisons |
| Min 35 samples discriminate between | **14** | comparisons |
| Min 40 samples discriminate between | **14** | comparisons |
| Min 45 samples discriminate between | **17** | comparisons |

| CS sulfation pattern | | | | |
|---|---|---|---|---|
| | **D0a0** | **D0a4** | **D0a6** | **D0a10** |
| **Adjacent** | 68 | 29 | 60 | 19 |
| **Cirr_HBV** | 94 | 33 | 499 | 94434 |
| **Cirr_HCV** | 41 | 13 | 264 | 22 |
| **Cirr_PSC** | 6611 | 62 | 236 | 61 |
| **HCC_ALDC** | 17 | 19 | 32 | 17 |
| **HCC_HBV** | 10 | 25 | 8 | 147 |
| **HCC_HCV** | 6 | 13 | 6 | 240 |

| | | |
|---|---|---|
| Min 20 samples discriminate between | **10** | comparisons |
| Min 25 samples discriminate between | **12** | comparisons |
| Min 30 samples discriminate between | **13** | comparisons |
| Min 35 samples discriminate between | **15** | comparisons |
| Min 40 samples discriminate between | **15** | comparisons |
| Min 45 samples discriminate between | **16** | comparisons |
